# RAB11A Promotes Cell Malignant Progression and Tumor Formation of Prostate Cancer via Activating FAK/AKT Signaling Pathway

**DOI:** 10.1155/2023/5885387

**Published:** 2023-01-31

**Authors:** Weifang Chen, Junjun Wang

**Affiliations:** ^1^Department of Hematology Oncology, Zhejiang Putuo Hospital, Zhoushan 316100, China; ^2^Department of Urology, Xiaoshan First Affiliated Hospital of Hangzhou Normal University, Hangzhou 311200, China

## Abstract

**Background:**

RAB11A, a member of the GTPase family, acts as a regulator in diverse cancers development. The dysregulation of the FAK/AKT signaling pathway is mainly related to tumorigenesis. This study aimed to investigate the possible effect of RAB11A in prostate cancer and further explore the potential mechanisms.

**Results:**

In this study, we illustrated the tumor-promoting effects of RAB11A based on *in vivo* and *in vitro* experiments. RAB11A expression was upregulated in prostate cancer cells. RAB11A knockdown decreased the prostate cancer cell proliferation, migration, and invasion. RAB11A also induced the epithelial-mesenchymal transition. PF562271 suppressed the malignant characteristics of prostate cancer cells caused by RAB11A knockdown. Furthermore, the interference of RAB11A reduced the tumor growth and the protein levels of p-FAK/FAK and p-AKT/AKT *in vivo*.

**Conclusion:**

RAB11A promotes cell malignant progression and tumor formation in prostate cancer via activating FAK/AKT signaling pathway.

## 1. Introduction

Prostate cancer is one of the most commonly diagnosed cancers in men worldwide, with approximately 191,930 cases and 33,330 deaths each year [1]. The incidence of prostate cancer is related to race and familial inheritance, especially in Africa and the USA [2]. Prostate cancer is usually diagnosed in the elderly (age >65 years) and ranks as the second cancer-related death in men [1]. Malignant transformation in the prostate is a multistage process that is initiated by localized prostate cancer, followed by adenocarcinoma with local invasion, and ends with metastatic prostate cancer [3]. Therapies for prostate cancer are mainly based on the pathologic evaluation of a prostate biopsy [4]. Surgery and radiotherapy are the main therapeutic approaches for early prostate cancer; however, they cannot effectively prevent tumor metastasis. Currently, the understanding of tumorigenesis mechanism of prostate cancer remains limited, which is an obstacle in the development of cancer therapies.

RAB11A is the first identified member of the small GTPase family, playing a crucial role in polarization during collective cell migration [5]. Previous studies found that RAB11A plays a critical role in cancer malignant progression through regulating the growth factor signaling [6, 7]. By activating of WNT signaling, RAB11A enhanced the proliferative capability and motility of esophageal cancer cells [8]. RAB11A is overexpressed in colorectal carcinoma and suppresses the expression of E-cadherin, which induces epithelial-mesenchymal transition (EMT) [9]. Besides, RAB11A is up-regulated in human gastric cancer cells and facilitates the proliferation and invasion of cancer cells through the FAK/AKT pathway [10]. These studies suggest RAB11A as an oncoprotein during cancer progression. However, the potential effects and mechanisms of RAB11A in prostate cancer remain unknown.

The focal adhesion kinase (FAK) signaling pathway is related to cell motility, which positively regulates tumorigenesis and metastasis [11]. FAK is upregulated in multiple cancers, such as gastric and prostate cancers, suggesting the deactivation of FAK signaling might be a possible approach for the treatment of cancer [10, 12]. FAK is an upstream target of ATP-dependent tyrosine kinase (AKT), and AKT acts as a migratory regulator in cancer cells [13]. Meanwhile, the activation of AKT could enhance the migration and invasion capabilities of cancer cells [14, 15]. Previous studies showed the FAK/AKT signaling pathway is activated in prostate cancer [12, 16]. Therefore, the FAK/AKT signaling pathway might be an effective target for cancer treatment, but its role in prostate cancer remains majorly elusive.

Therefore, the aim of the present study was to investigate the potential role of RAB11A in the development of prostate cancer. Meanwhile, the potential mechanism of RAB11A against prostate cancer was explored through *in vitro* and *in vivo* experiments to identify a novel potential target for the treatment of prostate cancer.

## 2. Materials and Methods

### 2.1. Cell Culture

A human prostatic epithelial cell line (RWPE1), purchased from the American Type Culture Collection (ATCC, Manassas, USA), was grown in defined keratinocyte serum-free medium (D-KSFM, Gibco, Carlsbad, CA, USA) at 37°C in 5% CO_2_ with saturated humidity. Prostate cancer cell lines (PC-3, VCaP, and DU145; ATCC, Manassas, USA) were cultured in minimum essential medium (MEM, Gibco, Carlsbad, CA, USA) with 10% fetal bovine serum (FBS, Gibco) in an incubator (Heracell 150i, Thermo Scientific, Carlsbad, CA, USA) at 37°C in 5% CO_2_ and saturated humidity. To prevent the contamination, the penicillin-streptomycin liquid (Solarbio, Beijing, China) was added to the medium.

### 2.2. Cell Transfection and Treatment

SiRNA targeting RAB11A (si-RAB11A) sequences were designed via the designer of small interfering RNA (DSIR, https://biodev.extra.cea.fr/dsir/dsir.php) (Table S1).

When the confluency of PC-3 cells was up to 70–90%, they were transfected with si-RAB11A, overexpressing (oe)-RAB11A, and empty vector (si-NC and oe-NC) plasmids (40 *μ*L, 1 × 10^8^ TU/mL). After transfection for 48 h, the medium was discarded, and complete medium with 2.5 *μ*g/mL puromycin was added to filter out the stable transfected cell line. The stable transfected cells were conducted for subsequent experiment. To analyze the effect of RAB11A on FAK/AKT signaling pathways, PC-3 cells were pretreated with 0.5 *μ*M PF562271 (a FAK inhibitor) for 24 h before the transfection.

### 2.3. Animal Model

Animal experimental procedures were approved by the Institutional Animal Care and Use Committee of Xiamen University (XMULAC20220034-1). A total of 10 male BALA/c nude mice, obtained from GemPharmatech Co. Ltd. (Nanjing, China), were cultured for 5-6 weeks, weighed 18−21 g, and were raised under specific nonpathogen conditions at 24−26°C and 40%–60% humidity. Mice were put into two groups at random (*n* = 5 each group): si-NC and si-RAB11A groups. After one week of adaptation, mice were subcutaneously injected with 3 × 10^6^ PC-3 cells transfected with si-NC or si-RAB11A. Ten days after transplantation, tumor sizes were detected every five days, and the volume was calculated: tumor volume (*v*) = 1/2 × length × (width^2^). After 30 days, mice were sacrificed via intraperitoneal injection with 160 mg/kg pentobarbital sodium, and death was considered as the asystole. Tumor tissues in each mouse were collected, weighed, and imaged.

### 2.4. Reverse Transcription-Quantitative PCR (RT-qPCR) Analysis

Total RNAs were extracted from cells and tumor tissues by Trizol (Invitrogen). cDNA was reversely transcribed using FastKing gDNA Dispelling RT Supermix (TIANGEN, KR118-02) and stored at −20°C. The SYBR Green PCR Master Mix (Lifeint, Guangzhou, China) was used to amplify cDNA in a cycler apparatus of the real-time system (MX3000P, Agilent Stratagene, California, USA). The thermocycling conditions were as follows: 95°C for 3 min, 40 cycles of annealing at 95°C for 12 s, and extension at 62°C for 40 s. The 2^−∆∆Ct^ method was employed to analyze the relative expression of genes. The composition of the RT-qPCR reaction mixture was listed at Table 1. Primer sequences used for RT-qPCR were presented in Table S2.

### 2.5. Western Blot Analysis

The transfected cell and tumor tissues lysing were accomplished with the radio immunoprecipitation assay buffer (RIPA, Beyotime, Shanghai, China) for total protein extraction. Protein concentration was determined using a bicinchoninic acid assay kit (BCA, Beyotime). Proteins were separated on 10% sodium dodecyl sulfate polyacrylamide gel electrophoresis (SDS-PAGE) by electrophoresis and transferred to polyvinylidene fluoride (PVDF) membrane. The membrane was blocked with 5% fat-free milk at room temperature for 1 h. Then, the membrane was incubated overnight at 4°C with primary antibodies, as listed at Table S3. Membranes were washed with TBST (a buffer combined with Tris buffered saline and Tween, Beyotime; 1 : 10) for 10 min thrice and incubated with secondary antibodies for 1 h. Finally, protein bands were visualized by the ECL reagents (Applygen, Beijing, China) and were quantified by Image J software.

### 2.6. Cell Counting Kit (CCK)-8 Assay

The cell viability assay was implemented with a CCK-8 assay kit (Beyotime). Briefly, transfected cells were digested by trypsin and then centrifuged at 1,000 g for 5 min to discard the supernatant. The fresh complete medium was used to suspend the cells. After suspension, cells (1 × 10^5^/mL) were seeded into 96-well plates with 40 *μ*L/well and incubated at 37°C with 5% CO_2_. After 24 h, each well was added with 10 *μ*L CCK-8 solution and incubated for 2 h. The absorbance value at 450 nm was measured by a microplate reader (DR-3518G, Wuxi Hiwell Diatek, Wuxi, China).

### 2.7. Colony Formation Assay

Cells (200 cells/well) were seeded into 6-well plates and cultured in 2 mL complete medium for 2 weeks. After 2 weeks of cell culture, the medium was discarded, and cells were rinsed with PBS twice. Following the fixation with 1 mL methanol for 15 min, cells were stained with crystal violet. Subsequently, the plate was reversely put on a white paper and then captured for further colony cell number counting by Image J software.

### 2.8. Wound Healing Assay

The cells in the logarithmic period were digested by trypsin for about 1 min, which was ended by the addition of complete medium. Then, the cells were centrifuged at 1,000 g for 5 min to discard the supernatant, and adjusted density with fresh complete medium. Cells (1 × 10^8^ cells/well) were seeded in 6-well plates with complete medium. After 24 h incubation, cells were scratched with pipette tip, washed with PBS three times, and then put into a chamber at 37°C with 5% CO_2_. Cell migration was observed between 0 and 24 h. The cell migration at 0 h was captured as a control for further comparison with the migration condition at 24 h.

### 2.9. Transwell Invasion Assay

The Transwell chamber was pretreated with 50 *μ*L Matrigel (50 mg/L, dilution: 1 : 4) and incubated at 37°C for 2 h. After digestion by trypsin, cells were washed and resuspended by PBS to a cell density of 1 × 10^6^/mL. The cells were suspended in serum-free medium. Upper and lower chambers of Transwell were added with 200 *μ*L cell suspension and 600 *μ*L medium with 10% FBS, respectively. Following incubation at 37°C with 5% CO_2_ for 24 h, the medium was discarded, and cells were fixed with formaldehyde for 30 min. After fixation, cells were stained with 0.1% crystal violet for 20 min, and imaged three random regions under a microscope (SC180, Olympus, Japan).

### 2.10. Statistical Analysis

Data are presented as mean ± standard deviation. GraphPad Prism 7.0 software (GraphPad software, USA) was employed to analyze all experimental data. A one-way ANOVA was applied to compare data obtained from multiple groups. Statistical significance was regarded as *P*  <  0.05.

## 3. Results

### 3.1. RAB11A Expression is Upregulated in Prostate Cancer Cells

**Figure 1:**
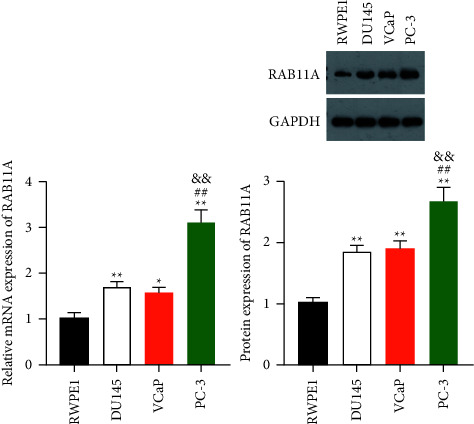
RAB11A expression is up-regulated in prostate cancer cells. The mRNA and protein expression of RAB11A in human prostate cancer cell lines (DU145, VCaP, and PC-3) and normal RWPE1 cell lines were detected by RT-qPCR and western blotting, respectively. ^*∗*^*P*  <  0.05, ^*∗∗*^*P*  <  0.01 vs. RWPE1; ## *P*  <  0.01 vs. DU145; && *P*  <  0.01 vs. VCaP.

RAB11A is a crucial regulator in multiple cancers. We first used RT-qPCR and western blot assays to measure the expression level of RAB11A in prostate cancer cells. Results showed both the mRNA and protein expression of RAB11A were remarkably overexpressed in human prostate cancer cell lines (DU145, VCaP, and PC-3) compared with those in normal RWPE1 cells (*P*  <  0.05). Notably, the expression level of in PC-3 cells was relatively highest (*P*  <  0.01) (Figure 1).

### 3.2. RAB11A Promotes Prostate Cancer Cell Proliferation

**Figure 2:**
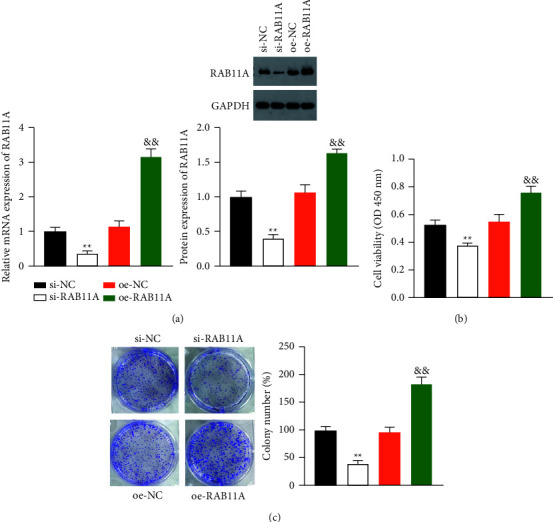
RAB11A promotes prostate cancer cell proliferation. (a) The mRNA and protein levels of RAB11A in PC-3 cells were detected by RT-qPCR and western blotting, respectively. (b) The viability of PC-3 cells was analyzed by the CCK-8 assay. (c) The proliferation of PC-3 cells was examined by a clone formation assay. PC-3 cells were transfected with si-NC, si-RAB11A, oe-NC, or oe-RAB11A. ^*∗∗*^*P*  <  0.01 vs. si-NC; && *P*  <  0.01 vs. oe-NC.

Since RAB11A expression was obviously higher in PC-3 cells relative to normal cells, we examined the effects of RAB11A on PC-3 by transfecting PC-3 cell lines with siRNA-RAB11A (si-RAB11A), overexpress RAB11A (oe-RAB11A), and a negative control (NC). The mRNA and protein levels of RAB11A were reduced by si-RAB11A and enhanced by oe-RAB11A (*P*  <  0.01) (Figure 2(a)). CCK-8 and colony formation assays showed that, compared to the NC groups, the interference of RAB11A remarkably reduced the proliferation of PC-3 cells, and RAB11A overexpression enhanced cell proliferation (*P*  <  0.01) (Figures 2(b) and 2(c)).

### 3.3. RAB11A Facilitates the Migration, Invasion, and Epithelial-Mesenchymal Transition (EMT) of Prostate Cancer Cells

**Figure 3:**
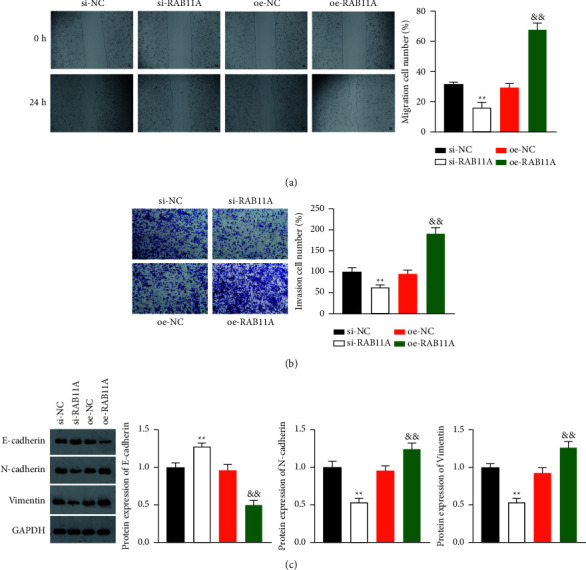
RAB11A promotes the migration and invasion of prostate cancer cells. (a) Cell migration ability was detected by wound healing assay (scale bar = 50 *μ*m); (b) Cell invasion ability was detected by Transwell assay (scale bar = 50 *μ*m); (c) The expressions of E-cadherin, N-cadherin, and vimentin in PC-3 cells were detected by western blot analysis. PC-3 cells were transfected with si-NC, si-RAB11A, oe-NC, or oe-RAB11A. ^*∗∗*^*P*  <  0.01 vs. si-NC; && *P*  <  0.01 vs. oe-NC.

Next, we determined the influence of RAB11A on the invasion and migration of cancer cells. Wound healing assay demonstrated the decreased migratory potential of PC-3 cells with RAB11A knockdown and the increased migration with RAB11A overexpression (*P*  <  0.01) (Figure 3(a)). Transwell assay suggested that RAB11A overexpression markedly enhanced cell invasion, while RAB11A knockdown presented the opposite effect (*P*  <  0.01) (Figure 3(b)). Subsequently, we used western blot assay to detect the levels of E-cadherin, N-cadherin, and vimentin. Results revealed that the knockdown of RAB11A, compared with the NC group, enhanced the level of the epithelial marker E-cadherin but suppressed the expression of mesenchymal markers N-cadherin and vimentin; meanwhile, RAB11A overexpression presented opposite effects (*P*  <  0.01) (Figure 3(c)).

### 3.4. RAB11A Promotes Proliferation, Migration, Invasion, and EMT of Prostate Cancer Cells via Activating FAK/AKT Signaling Pathway

**Figure 4:**
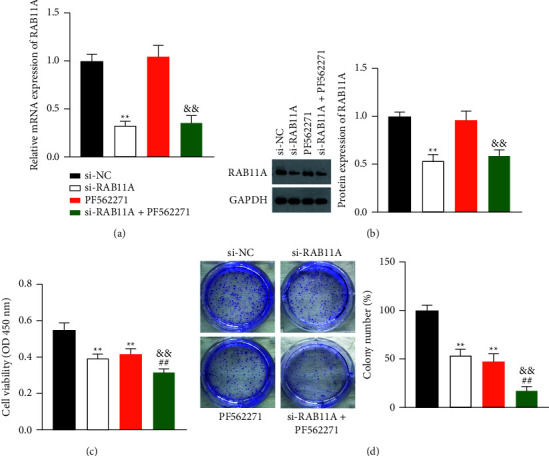
RAB11A promotes proliferation of prostate cancer cells via the activating FAK/AKT signaling pathway. (a) The mRNA expression of RAB11A in PC-3 cells was detected by RT-qPCR. (b) The protein expression levels of RAB11A in PC-3 cells were detected by western blotting. (c) PC-3 cell viability was analyzed by CCK-8 assay. (d) The proliferation of PC-3 cells was examined by clone formation assay. The PC-3 cells were pretreated with the FAK inhibitor PF562271 before the transfection with si-RAB11A. ^*∗∗*^*P*  <  0.01 vs. si-NC; ^##^*P*  <  0.01 vs. si-RAB11A; && *P*  <  0.01 vs. PF562271.

**Figure 5:**
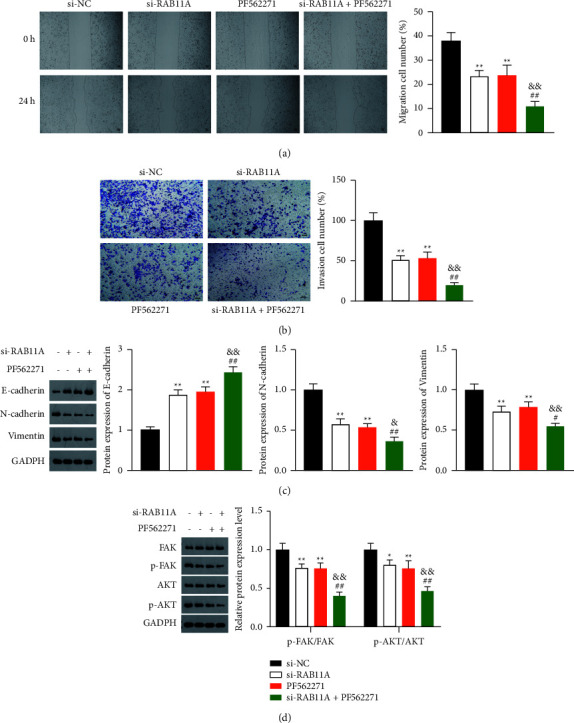
RAB11A promotes migration, invasion, and EMT of prostate cancer cells via activating the FAK/AKT signaling pathway. (a) The migration of PC-3 cells was analyzed by wound healing assay. (b) The invasion of PC-3 cells was analyzed by Transwell assay (scale bar = 50 *μ*m). (c) The protein levels of E-cadherin, N-cadherin, and vimentin were analyzed by western blot analysis. (d) The protein levels of p-FAK/FAK and p-AKT/AKT were analyzed by western blot analysis. The PC-3 cells were pretreated with the FAK inhibitor PF562271 before the transfection with si-RAB11A. ^∗^*P* < 0.05, ^∗∗^*P* < 0.01 vs. si-NC; ^#^*P* < 0.05, ^##^*P* < 0.01 vs. si-RAB11A; &*P* < 0.05, &&*P* < 0.01 vs. PF562271.

Activation of FAK/AKT promotes prostate cancer cell aggression. Thus, we hypothesized that the potential mechanism of RAB11A in prostate cancer might be associated with the FAK/AKT signaling pathway. To verify this, we pretreated PC-3 cells with the FAK inhibitor PF562271 before transfection with si-RAB11A. Results showed that PF562271 did not affect the RAB11A expression level in transfected PC-3 cells (Figures 4(a) and 4(b)). Besides, CCK-8 and clone formation assays demonstrated that PF562271 remarkably reduced the proliferative capability of the PC-3 cells with si-RAB11A (*P*  <  0.01) (Figures 4(c) and 4(d)). Meanwhile, wound healing and Transwell experiments revealed that the migration and invasion of PC-3 cells with si-RAB11A were remarkably inhibited by PF562271 addition (*P*  <  0.01) (Figures 5(a) and 5(b)). In addition, western blot experiment demonstrated that PF562271 suppressed the expression of N-cadherin and vimentin and increased E-cadherin expression in PC-3 cells transfected with si-RAB11A (*P*  <  0.01) (Figure 5(c)). Furthermore, RAB11A knockdown decreased the protein expression of p-FAK/FAK and p-AKT/AKT, and PF562271 addition enhanced the inhibitory effect of si-RAB11A on the FAK/AKT pathway in PC-3 cells (*P*  <  0.01) (Figure 5(d)).

### 3.5. RAB11A Promotes the Tumor Formation of Prostate Cancer via Activating FAK/AKT Signaling *In Vivo*

**Figure 6:**
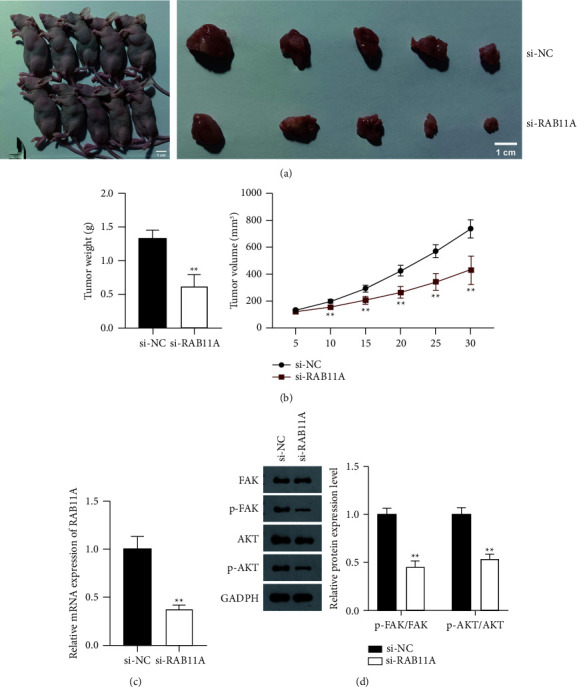
RAB11A promotes the tumor formation of prostate cancer via activating the FAK/AKT signaling pathway *in vivo*. (a) Tumor size (scale bar = 1 cm); (b) tumor weight and volume. (c) The mRNA expression of RAB11A in tumor tissues of mice was detected by RT-qPCR. (d) The protein levels of p-FAK/FAK and p-AKT/AKT in tumor tissues were measured by western blotting. Mice were subcutaneously injected with PC-3 cells that were transfected with si-NC or si-RAB11A. After 30 days transfection, mice were euthanized, and tumor tissues were collected. ^*∗∗*^*P*  <  0.01 vs. si-NC.

To evaluate the effects and mechanism of RAB11A in prostate cancer *in vivo*, a xenograft mouse tumor model was created by transfecting with si-RAB11A. The images of euthanized mice and the sizes of collected tumors are presented in Figure 6(a), showing the absence of RAB11A reduced tumor sizes. The weight and volume of tumor tissues in the si-RAB11A group were significantly lower than si-NC group (*P*  <  0.01) (Figure 6(b)). RT-qPCR showed that RAB11A expression, compared with the si-NC group, was remarkably decreased by si-RAB11A (*P*  <  0.01) (Figure 6(c)). On the other hand, the protein levels of FAK, AKT, p-FAK, and p-AKT were detected by western blot assay. Results showed the p-FAK/FAK and p-AKT/AKT protein levels are obviously decreased in si-RAB11A group (*P*  <  0.01) (Figure 6(d)).

## 4. Discussion

Prostate cancer is the secondary diagnosed malignancy in men worldwide, with high mortality. The etiology of prostate cancer remains elusive because of the lacked evidence of genetic and pathology [17]. Previous studies showed there are several signaling pathways were mainly involved in prostate cancer, including MEK/ERK, FAK/AKT, and p75NTR signaling pathway [12, 18, 19]. Risk factors regulating these signaling pathways could be regarded as a potential target in the treatment of prostate cancer. RAB11A is overexpressed in diverse types of human cancers, such as lung and gastric cancer [6, 10]. In this work, we found that the RAB11A is upregulated in prostate cancer cells and promotes the progression of prostate cancer *in vitro* and *in vivo* through activating FAK/AKT signaling.

RAB11A is a major GTPase of vesicular trafficking and membrane dynamics, the alterations of which promote tumorigenesis [20]. It has been reported that RAB11A facilitates gastric cancer progression and metastasis [10]. Our data showed a similar effect of RAB11A overexpression in prostate cancer, promoting the proliferation, invasion, and migration of cancer cells. Meanwhile, the RAB11A expression was positively related with mesenchymal markers (N-cadherin and vimentin) but negatively with epithelial marker E-cadherin. These results demonstrate that RAB11A plays an oncological role in prostate cancer.

FAK is an important regulator of cell migration and invasion [21, 22]. FAK signaling pathway was activated in multiple human cancers including prostate cancer [16]. AKT is activated by FAK stimulation, regulating cell migration and invasion [23]. Accumulating evidence suggests that the activation of FAK/AKT signaling promotes tumorigenesis. CCK3 contributes to the EMT process in prostate cancer by activating FAK/AKT signaling [12]. Knockdown of RABL3, which belongs to the Rab subfamily, inhibits the proliferation and invasion of oral squamous cell carcinoma through deactivating the FAK/AKT pathway [24]. As another member of the Rab subfamily, RAB11A in the present study showed similar functions, facilitating the proliferation, migration, and invasion of prostate cancer. Therefore, we speculated that RAB11A promotes prostate cancer progression via the FAK/AKT pathway. Our data showed that si-RAB11A reduced the expression of p-FAK/FAK and p-AKT/AKT. PF562271 (a FAK inhibitor) enhanced the inhibitory effect of RAB11A on the FAK/AKT signaling pathway and on the malignant progression of prostate cancer. These results demonstrate that RAB11A could potentially promote the malignant progression of prostate cancer by activating the FAK/AKT signaling pathway. Meanwhile, *in vivo* experiment showed that the interference of RAB11A reduced the tumor growth and downregulated the protein levels of p-FAK/FAK and p-AKT/AKT in tumor tissues of prostate cancer. Taken together, our data suggest that RAB11A, as an oncogenous protein, promotes prostate cancer malignant progression and tumorigenesis through activating FAK/AKT signaling.

In conclusion, the current study identified the role of RAB11A as a tumor promoter overexpressed in human prostate cancer. The possible mechanism of RAB11A promoting prostate cancer is associated with the activation of the FAK/AKT pathway. Meanwhile, this study suggests the FAK/AKT signaling pathway as a therapeutic target to regulate the progression and development of prostate cancer. However, the understanding of the underlying intermediate mechanism by which RAB11A regulates the FAK/AKT signaling pathway remains unrevealed.

## Figures and Tables

**Table 1:** The composition of the RT-qPCR reaction mixture.

Components	Volume (*μ*L)
2 × mixture	10 *μ*L
PCR forward primer (10 *μ*M)	1 *μ*L
PCR reverse primer (10 *μ*M)	1 *μ*L
cDNA template	1 *μ*L
ddH_2_O	7 *μ*L
Total	Up to 20 *μ*L

## Data Availability

The data used to support the findings of this study are available from the corresponding author upon request.

## Supplementary Materials

Supplementary Materials Table S1: the sequence of si-RAB11A. Table S2: primer sequences used for RT-qPCR. Table S3: the information of antibodies.
