# Supplementary material for: RAB11A Promotes Cell Malignant Progression and Tumor Formation of Prostate Cancer via Activating FAK/AKT Signaling Pathway
Source: Evid Based Complement Alternat Med. 2023 Jan 31;2023:5885387. doi: 10.1155/2023/5885387 (PMC9904921; doi:10.1155/2023/5885387)
Supplement: Supplementary Materials — Table S1: the sequence of si-RAB11A. Table S2: primer sequences used for RT-qPCR. Table S3: the information of antibodies. [file 5885387.f1.zip › Table S1.doc]

Table S1: The sequence of si-RAB11A.

| Name |  | Sequence (5′-3′) |
| --- | --- | --- |
| si-RAB11A (Human) | SS Sequence | GCGACGACGAGUACGACUACC |
|  | AS Sequence | UAGUCGUACUCGUCGUCGCGG |
