# Supplementary material for: RAB11A Promotes Cell Malignant Progression and Tumor Formation of Prostate Cancer via Activating FAK/AKT Signaling Pathway
Source: Evid Based Complement Alternat Med. 2023 Jan 31;2023:5885387. doi: 10.1155/2023/5885387 (PMC9904921; doi:10.1155/2023/5885387)
Supplement: Supplementary Materials — Table S1: the sequence of si-RAB11A. Table S2: primer sequences used for RT-qPCR. Table S3: the information of antibodies. [file 5885387.f1.zip › Table S2.doc]

Table S2: Primer sequences used for RT-qPCR.

| Name | Sequence (5′-3′) | Length (bp) |
| --- | --- | --- |
| Human RAB11A forward | ACGTCATCTCAGGGCAGTTC | 94 |
| Human RAB11A reverse | TGTAGAGTCTAGGGCCGAAGT |  |
| Human GAPDH forward | TGTGGGCATCAATGGATTTGG | 116 |
| Human GAPDH reverse | ACACCATGTATTCCGGGTCAAT |  |
| Mouse RAB11A forward | ACGTCATCTCAGGGCAGTTC | 241 |
| Mouse RAB11A reverse | TGGCTTGTTCTCAGTGGTGG |  |
| Mouse GAPDH forward | TGTGAACGGATTTGGCCGTA | 71 |
| Mouse GAPDH reverse | CAATCTCCACTTTGCCACTGC |  |
