# Supplementary material for: RAB11A Promotes Cell Malignant Progression and Tumor Formation of Prostate Cancer via Activating FAK/AKT Signaling Pathway
Source: Evid Based Complement Alternat Med. 2023 Jan 31;2023:5885387. doi: 10.1155/2023/5885387 (PMC9904921; doi:10.1155/2023/5885387)
Supplement: Supplementary Materials — Table S1: the sequence of si-RAB11A. Table S2: primer sequences used for RT-qPCR. Table S3: the information of antibodies. [file 5885387.f1.zip › Table S3.doc]

Table S3: The information of antibodies.

| Name | CAS | Kind | Dilution |
| --- | --- | --- | --- |
| RAB11A | ab128913, Abcam, Cambridge, UK | Primary antibody | 1:10,000 |
| GAPDH | EPR16891, Abcam, | Primary antibody | 1:10,000 |
| N-cadherin | ab76011, Abcam, | Primary antibody | 1:5,000 |
| Vimentin | ab92547, Abcam, | Primary antibody | 1:1,000 |
| E-cadherin | ab40772, Abcam, | Primary antibody | 1:10,000 |
| FAK | ab76011, Abcam, | Primary antibody | 1:5,000 |
| AKT | 4685, CST, Boston, USA | Primary antibody | 1:1,000 |
| p-AKT | 4060, CST | Primary antibody | 1:2,000 |
| Goat anti-rabbit IgG | ab6721, CST | Secondary antibody | 1:2,000 |
